# Clinical Features and Factors Associated With Sepsis-Associated Encephalopathy in Children: Retrospective Single-Center Clinical Study

**DOI:** 10.3389/fneur.2022.838746

**Published:** 2022-05-16

**Authors:** Yihao Chen, Yan Hu, Xufeng Li, Peiling Chen, Chun Wang, Jing Wang, Jiaxing Wu, Yueyu Sun, Guilang Zheng, Yiyun Lu, Yuxiong Guo

**Affiliations:** ^1^Pediatric Intensive Care Unit, Guangdong Provincial People's Hospital, Guangdong Academy of Medical Sciences, Guangzhou, China; ^2^Guangdong Cardiovascular Institute, Guangdong Provincial People's Hospital, Guangdong Academy of Medical Sciences, Guangzhou, China; ^3^The Second School of Clinical Medicine, Southern Medical University, Guangzhou, China

**Keywords:** sepsis, sepsis-associated encephalopathy, children, risk factor (RF), retrospective study

## Abstract

**Background:**

Sepsis-associated encephalopathy (SAE) is a common complication in septic patients with a higher ICU and hospital mortality in adults and poorer long-term outcomes. Clinical presentation may range from mild confusion to convulsions and deep coma; however, little is known about SAE in children. We aimed to retrospectively analyze the data for children with sepsis, to illustrate the epidemiology, performance, and adverse outcome, and to evaluate the association between risk factors and SAE in children.

**Methods:**

All children with sepsis who were admitted to the Department of Pediatrics, Guangdong Provincial People's Hospital, Guangzhou, Guangdong, China from January 2010 to December 2020 were retrospectively analyzed.

**Results:**

A total of 210 patients with sepsis were retrospectively assigned to the SAE and non-SAE groups, of which 91 (43.33%) were diagnosed with SAE with a mortality of 6.70% (14/210). Significant differences were observed in the level of white blood platelet, platelets, international normalized ratio, prothrombin time, activated partial thromboplastin time, total protein, Ccr, UREA, blood urea nitrogen, alanine transaminase, aspartate transaminase, creatine kinase, creatine kinase isoenzymes, lactate dehydrogenase, procalcitonin, and lactic acid (*p* < 0.05). In the risk assessment scales, significant differences were observed in the modified Glasgow Coma score, PCIS, Pediatric Logistic Organ Dysfunction Score 2 (PELOD-2), Pediatric Sequential Organ Failure Assessment Score, and Pediatric Risk of Mortality III (*p* < 0.05). The incidence of septic shock, acute kidney disease, liver dysfunction, and coagulation disorder were higher in the SAE group (*p* < 0.05). The mechanical ventilation time ([6.57 d ± 16.86 d] *vs*. [2.05 d ± 5.79 d]; *p* < 0.001), CRRT time ([1.74 d ± 6.77 d] *vs*. [0.11 d ± 0.63 d]; *p* < 0.001), ICU stay time ([299.90 h ± 449.50 h] *vs*. [177.67 h ± 245.36 h]); *p* < 0.001 was longer than that of non-SAE. Both the PCT, Ca^2+^, septic shock, PELOD-2, and midazolam were identified as independent risk factors, and fentanyl was a protective factor for SAE in pediatric patients (*p* < 0.05). The main clinical neurological symptoms consisted of agitation, hypnosia, hypnosis alternates agitated, anterior fontanelle full/bulging/high tension, coma, muscle hypertonia, muscle hypotonia, hyperreflexia, focal seizure, and generalized seizure.

**Conclusions:**

The incidence of SAE in children was found high and the prognosis poor. In this retrospective study, the identified patients were more susceptible to SAE, with an inflammatory storm with hypocalcemia or septic shock. The use of midazolam will increase the occurrence of SAE, whereas fentanyl will reduce the incidence of SAE, and PELOD-2 may predict the occurrence of SAE.

## Introduction

Sepsis is defined as life-threatening organ dysfunction caused by a dysregulated host response to infection, which advances serious clinical consequences (e.g., septic shock and multiple organ dysfunction syndrome) (1, 2). Sepsis-associated encephalopathy (SAE) is a common complication in children with sepsis involving a diffuse brain disorder without clinical or laboratory data indicating a direct infection of the central nervous system. SAE can manifest as hypotonia, irritability, coma, cognitive impairment, disorientation, and focal neurological features (e.g., convulsions) (3–5). Furthermore, SAE can cause a severe unfavorable prognosis and develop permanent neurocognitive impairment (6–8). To date, the diagnostic criteria and risk factors for SAE remain in debate, whereas existing normative data have focused primarily on adults with relatively few studies examining childhood. Thus, the present study aimed to retrospectively analyze the data of children with sepsis, to illustrate the epidemiology, performance, adverse outcome, and risk factors for SAE.

## Methods

### Patients Selection

This was a retrospective study of all patients with sepsis who were admitted to the Department of Pediatrics, Guangdong Provincial People's Hospital, Guangzhou, Guangdong, China from January 2010 to December 2020. Patient inclusion criteria consisted of the following: (1) for the purpose of this study, we excluded the group of newborns because the special pathophysiology, growth, and development refer to children as those ranging from 28 days of age to 14 years; (2) sepsis will be defined as infection plus organ failure as per the Sepsis 3 criteria; (3) the standard for organ dysfunction in pediatrics was based on the International Pediatric Sepsis Consensus Conference in 2005 in the USA (9); (4) the diagnostic criteria for SAE were based on children with sepsis whose Glasgow Coma score <15 or progress noted clearly documented neuropsychiatric or cognitive disorders, including impaired concentration, delirium, disorientation, consciousness disorders, and epileptic seizures (10). Excluded were patients with (1) primary central nervous system diseases (e.g., intracranial infection, traumatic brain injury, cerebrovascular diseases, immune encephalitis, etc.); (2) an altered state of consciousness caused by metabolic disorders, such as hypoglycemia, hyperglycemia, hepatic encephalopathy, pulmonary encephalopathy, uremia encephalopathy, etc.; (3) genetic diseases that have an influence on neuropsychic behavior; (4) malignancies; (5) drug or toxic poisoning; and (6) an unclear prognosis.

### Data Collection and Processing

The following data were collected at the onset of sepsis or SAE in 24 h: (1) the baseline date of patients (e.g., sex, age, temperature, respiratory rate, heart rate, and mean artery pressure [MAP], the use of midazolam, and fentanyl); (2) risk assessment scale score: modified Glasgow Coma score (mGCS); Pediatric Critical Illness Score (PCIS); Pediatric Logistic Organ Dysfunction Score 2 (PELOD-2); Pediatric Sequential Organ Failure Assessment Score (p-SOFA); and Pediatric Risk of Mortality III (PRISMIII); (3) biochemical index (white blood cell [WBC] count; neutrophil percentage [N%]; hemoglobin [Hb]; hematocrit [HCT]; platelets [PLT]; international normalized ratio [INR]; prothrombin time [PT]; activated partial thromboplastin time [APTT]; fibrinogen [FIB]; D-Dimer [D-D]; total protein [TP]; albumin [ALB]; serum creatinine [Scr]; urea; blood urea nitrogen [BUN]; total bilirubin [TBIL]; alanine transaminase [ALT]; aspartate transaminase [AST]; creatine kinase [CK]; creatine kinase isoenzymes [CK-MB]; lactate dehydrogenase [LDH]; blood glucose [BG]; serum electrolyte; C-reactive protein [CRP]; procalcitonin [PCT]; and lactic acid [LAC]); (4) the clinical features between two groups (septic shock, acute kidney disease, liver dysfunction, and coagulation disorder), and patient outcomes (mechanical ventilation time, CRRT time, ICU stay time, length of stay and primary outcome); and (5) the clinical neurological symptoms and EEG features in SAE.

### Statistical Analysis

SPSS 22.0 (SPSS, Inc, NY, USA) was used for data analysis. The Kolmgorov–Smirnov test was used to assess the distribution of variables. Parametric continuous data were expressed as the mean ± standard deviation (SD), and non-parametric distribution was expressed as the median (interquartile ranges). Parametric continuous variables were compared using a *t*-test and non-parametric continuous variables with a Mann–Whitney U test. The chi-squared test was adopted to assess the differences in categorical variables between groups. The efficiency of the risk assessment scores to forecast imminent SAE was evaluated by the area under ROC curve (AUC). SAE-associated risk factors were identified via multivariate logistic regression analysis. Specifically, variables related to SAE in the univariate analysis (*p* < 0.1) were entered into a multivariate logistic regression analysis to calculate the estimated odds ratios (OR) and 95% confidence intervals (95%CI), in which the significant level for independent risk factors was *p* < 0.05.

## Results

### Baseline and Outcome Characteristics of the Two Patient Groups

**Figure 1:**
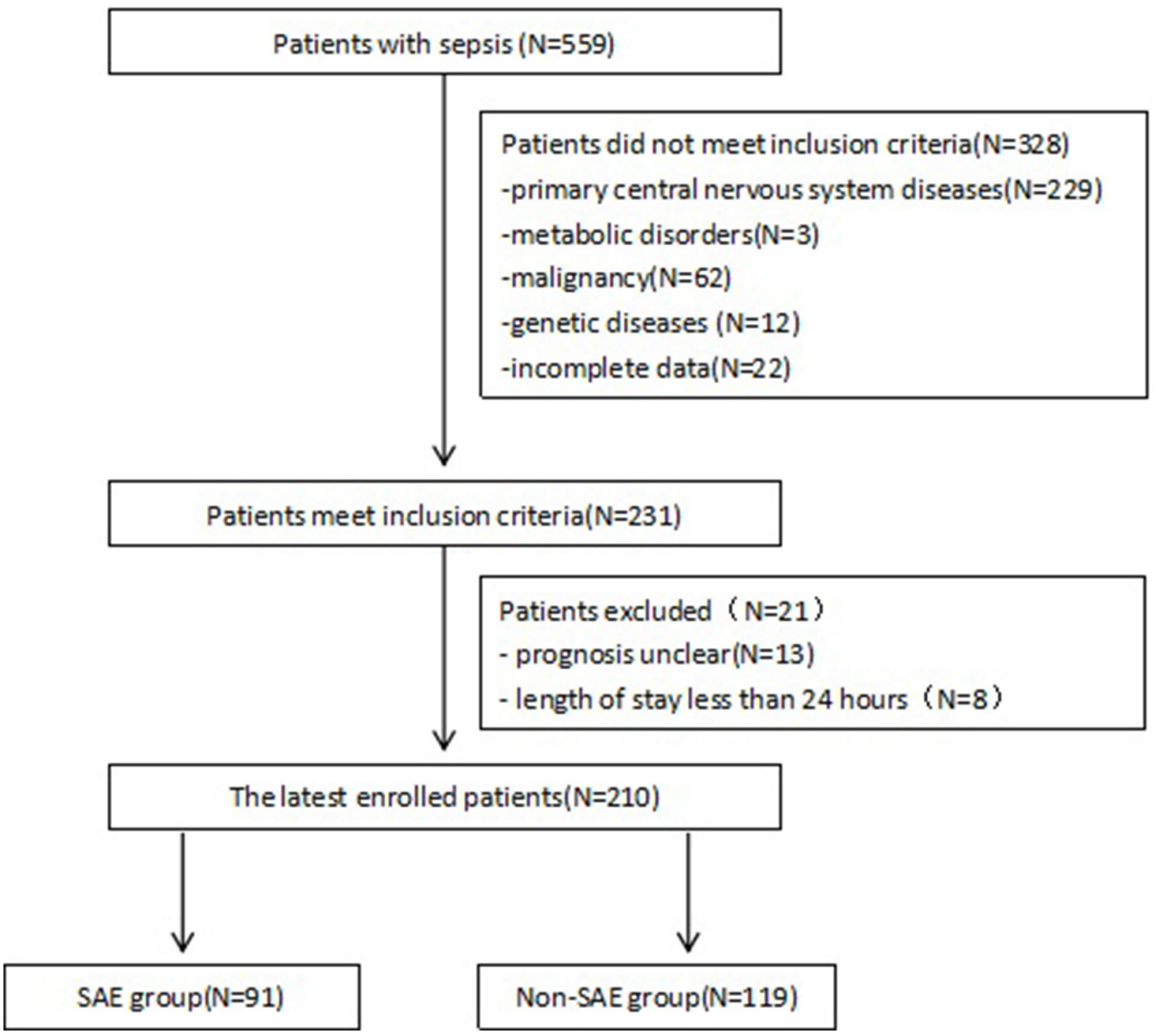
Study flow chart showing retrospective patient enrollment. SAE, Sepsis-associated encephalopathy.

After excluding 349 patients, a total of 210 patients with sepsis were retrospectively assigned to the SAE and non-SAE groups (Figure 1). Baseline and outcome characteristics are summarized in Table 1. There were 91 (43.3%; 57 males) patients who were diagnosed with SAE. The median ages of the SAE and non-SAE groups were 12.00 [4.00, 24.00] and 18.00 [7.00, 51.00] months old, respectively, with patients with SAE being significantly younger than patients with non-SAE (*p* = 0.001). In addition, the SAE group displayed a faster heart and respiratory rate (*p* < 0.05) and have a higher frequency of using midazolam and fentanyl (*p* < 0.001). The results are presented in Table 1.

**Table 1:** Patient baseline characteristics between two groups.

	**SAE group (*N* = 91)**	**Non-SAE group (*N* = 119)**	***p-*values**
**Age (months), median**	12.00 [4.00, 24.00]	18.00 [7.00, 51.00]	0.001 ^a^
**Sex** ***n*** **(%)**			
Male	57 (27.1)	67 (31.90)	0.355
Female	34 (16.20)	52 (24.80)	
**MAP (mmHg)**	59.04 ± 19.53	58.14 ± 27.15	0.780
**TEMP (** **°** **C)**	38.07 ± 1.29	37.61 ± 3.63	0.246
**Heart rate (bpm)**	151.22 ± 28.97	133.57 ± 29.35	*p* <0.001^a^
**Respiratory rate (bpm)**	36.00 [32.00, 47.00]	35.00 [25.00, 45.25]	0.022^a^
**Midazolam** ***n*** **(%)**	54 (25.7)	31 (14.8)	*p* <0.001^a^
**Fentanyl** ***n*** **(%)**	37 (17.6)	30 (14.3)	*p* <0.001^a^
**Risk assessment**			
mGCS	11.00 [5.00, 15.00]	15.00 [15.00, 15.00]	*p* <0.001^a^
PCIS	84.00 [78.00, 90.50]	90.00 [86.00, 96.00]	*p* <0.001^a^
PELOD-2	5.00 [2.75, 7.00]	2.00 [0.00, 2.50]	*p* <0.001^a^
p-SOFA	6.11 [3.00, 9.00]	2.62 [0.00, 4.00]	*p* <0.001^a^
PRISM III	7.00 [3.00, 12.00]	3.00 [0.00, 7.00]	*p* <0.001^a^

*SAE, Sepsis-associated encephalopathy; mGCS: modified Glasgow Coma score; PCIS, pediatric critical illness score; PELOD-2, pediatric logistic organ dysfunction score 2; p-SOFA, pediatric sequential organ failure assessment score; PRISMIII, pediatric risk of mortality III; a: P < 0.05*.

### Comparison of Biochemical Indexes, Clinical Features, and Outcomes Between Two Groups

The patients' biochemical indexes, clinical findings, and outcomes are listed in Tables 2, 3. We found that WBC, PLT, total protein, and serum calcium in the SAE group were significantly lower than that of the non-SAE group (*p* < 0.05). Moreover, INR, PT, and APTT were significantly longer (*p* < 0.05). Higher Scr, UREA, BUN, ALT, AST, CK, CK-MB, LDH, PCT, and LAC were observed in the SAE group (*p* < 0.05). Furthermore, these SAE patients had a higher incidence of septic shock, acute kidney disease, liver dysfunction, and coagulation disorder compared to their non-SAE counterparts. There was no significance in the between-group differences in other indicators (*p* > 0.05).

**Table 2:** Patient biochemical indexes between two groups.

	**SAE group (*N* = 91)**	**Non-SAE group (*N* = 119)**	***p-*values**
**Biochemical indexes**			
WBC (×10^9^/L)	10.99 [6.49, 15.94]	11.39 [8.20, 21.43]	0.016^a^
N (%)	66.60 [55.52, 84.13]	74.88 [48.93, 83.91]	0.464
HB (g/L)	95.00 [81.40, 106.00]	98.90 [86.00, 108.93]	0.24
HCT	0.28 ± 0.53	0.29 ± 0.54	0.96
PLT (×10^9^/L)	157.40 [71.00, 291.80]	234.90 [113.00, 424.23]	0.007^a^
INR	1.48 [1.12, 2.04]	1.17 [1.10, 1.32]	0.001^a^
PT (sec)	18.20 [14.50, 23.80]	14.80 [14.10, 16.25]	*p* <0.001^a^
APTT (sec)	45.40 [39.20, 66.60]	43.90 [36.67, 50.15]	0.009^a^
FIB (g/L)	3.00 [1.41, 4.44]	3.90 [2.29, 5.72]	0.054
D-D (ng/ml)	2610.00 [910.00, 6200.00]	1935.00 [665.00, 4040.00]	0.244
Total protein (g/L)	53.07 ± 10.96	57.25 ± 11.57	0.017^a^
Albumin (g/L)	28.56 ± 6.52	29.57 ± 7.59	0.321
Scr (umol/L)	43.00 [26.10,75.00]	30.15 [19.93,41.25]	0.01^a^
UREA (mmol/L)	5.50 [3.16, 12.71]	3.06 [2.06, 5.17]	0.031^a^
BUN (mg/dl)	15.40 [8.85, 35.59]	8.55 [5.76, 14.48]	0.036^a^
TBIL (umol/L)	13.30 [8.00, 22.40]	11.20 [6.05, 18.40]	0.092
ALT (U/L)	41.50 [22.25, 111.25]	23.00 [15.00, 41.50]	*p* <0.001^a^
AST (U/L)	77.00 [36.50, 178.30]	40.50 [29.25, 75.75]	0.001^a^
CK (U/L)	174.50 [49.50, 748.00]	89.80 [37.50, 184.25]	0.007^a^
CKMB (U/L)	25.70 [13.93, 52.60]	16.80 [9.93, 26.95]	*p* <0.001^a^
LDH (U/L)	543.25 [310.25, 1024.25]	354.00 [262.25, 607.25]	0.001^a^
Glucose (mmol/L)	5.55 [4.51, 7.03]	5.33 [4.63, 6.61]	0.501
Na (mmol/L)	134.00 [131.50, 137.48]	135.30 [132.75, 136.80]	0.724
K (mmol/L)	3.92 [3.34, 4.43]	3.84 [3.41, 4.32]	0.828
Ca (mmol/L)	2.10 [1.90, 2.20]	2.13 [2.00, 2.30]	0.025^a^
CRP (mg/L)	84.21 [31.25, 149.00]	52.20 [18.28, 131.48]	0.142
PCT (ng/ml)	11.23 [2.74, 40.00]	1.62 [0.48, 9.87]	*p* <0.001^a^
LAC (mmol/L)	1.45 [0.90, 2.83]	1.10 [0.80, 1.45]	0.015^a^

*WBC, white blood cell count; N%, neutrophil percentage; HB, hemoglobin; HCT, hematocrit; PLT, platelets; INR, international normalized ratio; PT, prothrombin time; APTT, activated partial thromboplastin time; TP, total protein; ALB, albumin; TBIL, total bilirubin; ALT, level of alanine aminotransferase; Scr, serum creatinine; BG, blood glucose; CRP, C-reactive protein; PCT, procalcitonin; LAC, lactic acid; a: p < 0.05*.

**Table 3:** Patients' clinical features between two groups.

	**SAE group** **(*N* = 91)**	**Non-SAE group** **(*N* = 119)**	***p-*values**
**Clinical findings** ***n*** **(%)**			
Septic shock	33 (15.70)	12 (5.70)	*p* <0.001^a^
Acute kidney disease	33 (15.70)	23 (11.00)	0.006^a^
Liver dysfunction	25 (11.90)	17 (8.10)	0.018^a^
Coagulation disorder	25 (11.90)	12 (5.70)	0.001^a^
**Outcome**			
Mechanical ventilation time (days)	6.57 ± 16.86	2.05 ± 5.79	*p* <0.001^a^
CRRT time (days)	1.74 ± 6.77	0.11 ± 0.63	*p* <0.001^a^
ICU stay time (hours)	299.90 ± 449.50	177.67 ± 245.36	*p* <0.001^a^
length of stay (days)	20.71 ± 20.82	16.96 ± 12.40	0.131
Mortality *n* (%)	14 (6.70)	6 (2.90)	0.039^a^

*CRRT time, continuous renal replacement therapy; ICU, Intensive Care Unit, a: p < 0.05*.

The outcome of the two groups included mechanical ventilation time, CRRT time, ICU stay time, length of hospital stay, and primary outcome. We found that the mechanical ventilation time ([6.57 d ± 16.86 d] *vs*. [2.05 d ± 5.79 d); *p* < 0.001], CRRT time [1.74 d ± 6.77 d] *vs*. [0.11 d ± 0.63 d]; *p* < 0.001), ICU stay time ([299.90 h ± 449.50 h] *vs*. [177.67 h ± 245.36 h]; *p* < 0.001) of patients with SAE was longer than that of the patients with non-SAE. There was no significant difference in the length of hospital stay. We concluded that the number of deaths in the SAE group was 14 compared to six in the non-SAE group, in which the mortality rate in the SAE group was 6.70%.

### Comparison of the Risk Assessment Scales Between the Two Patient Groups and ROC Curve Analysis for Predicting SAE

For the risk assessment scales, significant differences were observed between the two patient groups with respect to the PCIS, PELOD-2, p-SOFA, and PRISM III score (*p* < 0.05). The discrimination or the ability of the risk assessment scales to differentiate between the two groups is presented in Table 4. The ROC curve analysis showed that the AUCs of PCIS, PRISM III, PELOD-2, and p-SOFA for predicting the death of critically ill children were 0.723, 0.681, 0.808, and 0.769, respectively. Thus, PELOD-2 showed the best accuracy, followed by PCIS, PRISM III, and p-SOFA.

**Table 4:** Logistics analysis of the occurrence of sepsis-associated encephalopathy.

**Variable**	**OR**	**95%Cl**	***p*-values**
Ca^2+^	9.84	1.09, 88.55	0.041^a^
PELOD-2	1.41	1.16, 1.71	0.001^a^
Midazolam	13.55	2.43, 75.46	0.003^a^
Fentanyl	0.14	0.03, 0.80	0.027^a^
Septic shock	4.55	1.41, 14.69	0.011^a^
PCT	1.03	1.00, 1.05	0.027^a^

*PCT, procalcitonin; PELOD-2, Pediatric Logistic Organ Dysfunction Score 2, a: p < 0.05*.

### Multivariate Analysis of SAE Risk Factors in Sepsis

After adjusting for the baseline characteristics, biochemical indexes, risk assessment scores, and clinical features, the multivariate analysis revealed that the following independent risk factors for SAE in children were as follows (Table 5): use of midazolam (OR: 13.55, 95%CI: 2.43 − 75.46, *p* = 0.03), septic shock (OR: 4.55, 95%CI: 1.41 − 14.69, *P* = 0.11), PCT (OR: 1.03, 95%CI: 1.00 − 1.05, *p* = 0.279), Ca^2+^ (OR: 9.84, 95%CI: 1.09 − 88.56, *p* = 0.41), and PELOD-2 (OR: 1.41, 95%CI: 1.16 − 1.71, *p* = 0.01). The use of fentanyl is a protective factor for SAE (OR: 0.14, 95%CI: 0.03 − 0.80, *p* = 0.27).

**Table 5:** Predictive ability of PRISM III, PELID-2, p-SOFA, and PCIS on the occurrence of children with SAE.

**Score**	**AUC**	**Best cutoff**	**95%CI**	**Sensitivity (%)**	**Specificity (%)**	***p-*value**
PRISMIII^a^	0.681	6.5	0.604–0.757	54.90	74.30	<0.001
PELOD-2^a^	0.808	2.5	0.747–0.869	75.60	75.20	<0.001
P-SOFA^a^	0.769	3.5	0.703–0.836	70.70	71.60	<0.001
PCIS^a^	0.723	87	0.649–0.796	69.70	69.71	<0.001

*PCIS, Pediatric Critical Illness Score, PELOD-2, Pediatric Logistic Organ Dysfunction Score 2, p-SOFA, Pediatric Multiple Organ Dysfunction Score, PRISM III, Pediatric Risk of Mortality III score, a: p < 0.05*.

### Clinical Neurological Symptoms and Electroencephalogram (EEG) Features in SAE

The main clinical neurological symptoms of the 91 SAE cases are listed in Table 6, in which the common neurological symptoms were agitation (20 cases, 18.7%), hypnosia (26 cases, 24.3%), hypnosia alternates agitated (three cases, 2.8%); anterior fontanelle full/bulging/high tension (four cases, 3.7%); coma (11 cases, 10.3%); muscle hypertonia (four cases, 3.7%); muscle hypotonia (three cases, 2.8%); hyperreflexia (seven cases, 6.5%); focal seizure (four cases, 3.7%); and generalized seizure (25 cases, 23.3%). Retrospective research revealed that only 63 cases with SAE were detected with an EEG, and statistics and observations were analyzed (Table 7). Among the EEG in 63 cases, 12 were normal and 51 were abnormal. Abnormal EEG was most frequently associated with electrographic seizure (*n* = 30) (47.6%). They show a sharp wave, spike wave, sharp and slow wave complex, and epileptiform discharges on the EEG. An absence of reactivity was observed in 2 (3.2%) cases and periodic discharges in 4 (6.3%) cases. In addition, some children displayed an inappropriate delta and theta at their age in 15 (23.8%) cases.

**Table 6:** Clinical neurological symptoms of 91 cases of SAE.

**Clinical neurological symptoms**	**Cases**	**Percentages (%)**
Agitated	20	18.7
Hypnosia	26	24.3
Hypnosia alternates agitated	3	2.8
Anterior fontanelle full/bulging/high tension	4	3.7
Coma	11	10.3
Muscle hypertonia	4	3.7
Muscle hypotonia	3	2.8
Hyperreflexia	7	6.5
Focal seizure	4	3.7
Generalized seizure	25	23.3

*SAE, Sepsis-associated encephalopathy*.

**Table 7:** EEG features of 63 cases of SAE.

**EEG features**	**Cases**	**Percentages (%)**
**Normal EEG**	12	19.0
**Abnormal EEG**	51	81.0
Electrographic seizure	30	47.6
Absence of reactivity	2	3.2
Periodic discharges	4	6.3
inappropriate delta and theta at their age	15	23.8

*EEG, Electroencephalogram; SAE, Sepsis-associated encephalopathy*.

## Discussion

Due to a dysregulated host response to infection which can imperil all organ systems, the central nervous system is susceptible to SAE when affected by inflammation, oxidation, immunity, etc. (3, 11). SAE can occur at any stage of sepsis, which is one of the main manifestations of organ dysfunction with an incidence of 30–70% (12, 13). SAE is considered to be a component of poor clinical prognosis during sepsis and is typically associated with the mechanical ventilation time and, length of ICU stay, for which mortality was as high as 70% (14). In our study, the incidence of SAE in pediatric patients was 35.9% and the mortality was 6.70%, which was similar to the results of adults. Therefore, the risk factors in our research are of great significance for early clinical identification and active intervention to reduce the incidence and mortality in children with SAE.

The main clinical neurological symptoms of these cases included: agitation (20 cases, 18.7%), hypnosia (26 cases, 24.3%); hypnosia alternates agitated (three cases, 2.8%); anterior fontanelle full/bulging/high tension (four cases, 3.7%); coma (11 cases, 10.3%); muscle hypertonia (four cases, 3.7%); muscle hypotonia (three cases, 2.8%); hyperreflexia (seven cases, 6.5%); focal seizure (four cases, 3.7%); and generalized seizure (15 cases, 23.3%). The neurological symptoms were both similar to that of previous reports (15); however, they suggest that besides knowing some common symptoms, clinicians should strengthen the clinical observations and look for clues in the early stages of the disease among the symptoms of anterior fontanelle tension, muscular tension, and reflexivity.

The pathophysiology of SAE remains incompletely understood. The most recent studies have reported that multiple factors are involved in the pathophysiology of SAE, including neuroendocrine network disorders, inflammatory cytokine release, blood-brain barrier destruction, vascular function impaired, and neurotransmitter imbalance (15). In our analysis, PCT and Lac were found to be significantly increased, while PLT, total protein, and serum calcium were significantly decreased in the SAE group. Moreover, the incidence of septic shock, acute kidney disease, liver dysfunction, and coagulation disorder were significantly higher in the SAE group compared to that in the non-SAE group. PELOD-2 displayed the greatest accuracy for predicting SAE in the risk assessment scales.

Neuroinflammation, which represents one of the reasons for massive brain cell apoptosis, including microglia, neurons, and endothelial cell, plays a crucial role in the pathogenesis of SAE (16–18). Endotoxin causes an inflammatory response, which stimulated the production of proinflammatory cytokines, including IL-1, IL-6, or TNF-α. These cytokines have an influence on the brain and promote the synthesis of nitric oxide (NO), thereby affecting the neuro-endocrine network (19–21). As an early marker of systemic inflammation, the level of PCT is associated with bacterial endotoxins and pro-inflammatory cytokines (22), which was correlated with the severity of sepsis (19, 23). In the present study, we found that PCT was an independent risk factor for SAE.

At the same time, an early event in the inflammatory response is endothelium dysfunction, and the exposure of the subendothelial components of blood vessels will cause platelet over-activation. Activated platelets have key thromboinflammatory functions, which mediate coagulation and the immune response (24, 25). Following activation of the early coagulation process, a large number of microthrombi were formed, platelets, and procoagulants, and anticoagulant substances were further consumed, leading to microcirculatory disorders, resulting in tissue ischemia and hypoxia (26, 27). In addition, hemodynamics were impacted by microcirculatory alterations, resulting in lactic acid accumulation. One study showed that the brain functional capillary density and proportion of small, perfused vessels were significantly reduced at the onset of septic shock (28). The progressive loss of cerebral blood flow regulation might be associated with cortical dysfunction (29). Since the brain tissue has the lowest tolerance to hypoxia, children with sepsis who have thrombocytopenia, coagulation disorder, and septic shock are susceptible to SAE. Our analysis revealed that septic shock was an independent risk factor for SAE in children.

Among the children with sepsis, the stress state not only increased protein decomposition, but also increased the release of inflammatory cytokines, vascular endothelial cell damage, microcirculation disorders, aggravated capillary leakage, liver dysfunction, and liver protein synthesis ability decline, which resulted in a reduction in the total serum protein, especially albumin (30, 31). However, no significant difference was observed regarding albumin, which may be related to all children with sepsis exhibiting hypoalbuminemia. In addition, several studies have shown that the accumulation of neurotoxic substances was associated with renal insufficiency, which is one of the risk factors for SAE (4, 7).

Ca^2+^ is the main bivalent cation in the extracellular fluid, which plays an important role in neuromuscular conduction, muscle contraction, and cell membrane stability. Sepsis patients are prone to hypocalcemia, which is associated with a poor prognosis (32–34). Ca^2+^ was found to be significantly associated with high levels of proinflammatory cytokines (e.g., TNF-α and IL-6) and procalcitonin in severe sepsis (35). In addition, *Ca*^2+^ is a ubiquitous intracellular messenger or coenzyme that controls diverse cellular functions. Acetylcholine regulates cerebral blood flow through *Ca*^2+^dependent channels by stimulating brain microvascular endothelial cells to produce NO (36, 37). The nerve conduction function requires normal *Ca*^2+^ concentrations (38). Increases in ROS and Ca^2+^ play a critical role in neuronal excitation and glutamate, the primary excitatory neurotransmitter in the human brain, activates AMPK in cortical neurons (39). Several studies have shown hypocalcemia to be associated with SAE (40–42), and our present study revealed that it is an independent risk factor. EEG in hypocalcemia is characterized by generalized theta/delta range background slowing and focal or generalized spike and wave discharges, suggesting central neural hyperexcitability (43). It is further speculated that in sepsis with hypocalcemia, there is both increased neuromuscular excitability, which causes convulsions, as well as reduced cerebral blood flow and abnormal nerve conduction function, which causes cerebral ischemia-hypoxia and finally promotes the occurrence of SAE.

Sedation and analgesia are important treatments for critically ill patients, whereas excessive sedation is associated with delirium among patients who require longer mechanical ventilation and prolonged ICU stay (44). Our research suggests that while midazolam is an independent risk factor for SAE, a recent study of non-cardiac surgery in elderly patients revealed that the early postoperative use of midazolam was not associated with early delirium, which may be caused by age bias (45). Although analgesia as the basis of sedation may reduce sedative doses, our findings suggest that analgesics are a protective factor in SAE. A recent study showed that emergency surgery, high-dose midazolam, and fentanyl may be independent risk factors for SAE in mechanically ventilated patients (44). There are also conflicting data regarding analgesic effects. The study by Pandhairpande et al. reasoned that fentanyl was a risk factor for delirium in both surgical and traumatic ICU patients (46), whereas Agarwal et al. showed that the use of fentanyl reduced the occurrence of delirium (47). Moreover, another study showed that remifentanil combined with midazolam was an independent protective factor for delirium, with an incidence of 57.1% in patients without midazolam alone (48). The above contradictory data may indicate the depth of sedation and intensity of the analgesia may affect the functional state of the brain, which should be further demonstrated by future prospective studies.

For the risk assessment scales, PELOD-2 displayed the best accuracy, followed by PCIS, PRISM III, and p-SOFA. A prospective study revealed that the scores for PICU on days 1, 2, 5, 12, and 18, as well as on the day of discharge, PELOD-2 showed the progression of organ dysfunction severity and provides useful information for the prognosis of critically ill children (49). Our research suggests that PELOD-2 is an independent risk factor for SAE. It further confirmed the advantages of PELOD-2 in assessing the condition of critically ill patients.

### Limitations

There are some limitations associated with this study. First, since SAE is a potentially reversible syndrome, a single, point-in-time observation is not likely an accurate reflection of the actual neurological condition of critically ill patients. A dynamic assessment of risk factors may contribute to a better prognostic judgment of SAE. Second, the outcome of SAE may be associated with the severity of the primary disease. Future research should expand the sample quantity to assess the correlation. Finally, this was a retrospective study with minimal imaging data for children with SAE, future studies should add more objective indexes, including a prospective study utilizing continuous EEG monitoring to help identify SAE more accurately and rapidly.

## Conclusion

The incidence of SAE in children is high and the prognosis is poor. In this retrospective study, the identified patients more susceptible to SAE were associated with an inflammatory storm with hypocalcemia or septic shock. The use of midazolam will increase the occurrence of SAE, whereas fentanyl will reduce SAE, and PELOD-2 may predict the occurrence of SAE. Therefore, it is necessary to improve the awareness of SAE and reduce the risk factors to decrease the incidence of SAE.

## Data Availability Statement

The raw data supporting the conclusions of this article will be made available by the authors, without undue reservation.

## Ethics Statement

The studies involving human participants were reviewed and approved by the Medical Ethics Committee of Guangdong Provincial People's Hospital, Guangdong Academy of Medical Sciences. Written informed consent from the participants' legal guardian/next of kin was not required to participate in this study in accordance with the national legislation and the institutional requirements.

## Author Contributions

YC: writing original draft. YH, CW, XL, PC, JWu, YC, GZ, and YS: data collection. YH, YG, and YL: writing review. XL and YC: statistical analysis. CW, YC, GZ, and YS: experimental design. CW, XL, PC, JWu, YC, GZ, and YS: table design. YG and YL: project administration and supervision. All authors contributed to the article and approved the submitted version.

## Funding

This study was funded by Medical Scientific Research Foundation of Guangdong Province of China (Grant No. A2021460) and Matching funds for Distinguished Young Medical Talents of Climbing Program in Guangdong, China (Grant No. KJ012019451).

## Conflict of Interest

The authors declare that the research was conducted in the absence of any commercial or financial relationships that could be construed as a potential conflict of interest.

## Publisher's Note

All claims expressed in this article are solely those of the authors and do not necessarily represent those of their affiliated organizations, or those of the publisher, the editors and the reviewers. Any product that may be evaluated in this article, or claim that may be made by its manufacturer, is not guaranteed or endorsed by the publisher.
